# Building the skills and confidence of early childhood educators to work with parents: study protocol for the *Partnering with Parents* cluster randomised controlled trial

**DOI:** 10.1186/s12874-019-0846-1

**Published:** 2019-10-24

**Authors:** Zvezdana Petrovic, Olivia Clayton, Jan Matthews, Catherine Wade, Lina Tan, Denny Meyer, Antony Gates, Alex Almendingen, Warren Cann

**Affiliations:** 1 Parenting Research Centre, 5/232 Victoria Parade, East Melbourne, Victoria 3002 Australia; 2 Department of Statistics, Data Science and Epidemiology, Swinburne University of Technology, John Street, Hawthorn, Victoria 3122 Australia

**Keywords:** Cluster randomised controlled trial, Early childhood education, Practice model, Parents, Educators, Confidence, Partnership

## Abstract

**Background:**

In the early years of life, the benefits of parental engagement in children’s learning are well documented. Early childhood educators are a potentially effective source of support, having opportunity to engage with parents on key issues related to children’s learning and development. Educators report a need for more practical strategies for building positive partnerships with the parents of children in their care. To address this need, we have developed a practice support system, *Partnering with Parents*, to guide educators in Early Childhood Education and Care (ECEC) through practical strategies for working with parents. *Partnering with Parents* is designed to be embedded in everyday service delivery.

**Methods:**

Using a cluster randomised controlled trial (cRCT) with intervention and wait-list control groups, we aim to evaluate the effectiveness of the *Partnering with Parents* practice support system under normal service conditions. The intervention is being trialled in ECEC services across Victoria, Australia. Services in the intervention group implemented the 10-week intervention before the control group commenced the intervention. Educators and parents of children attending the participating services are taking part in evaluating the intervention by completing questionnaires online at three time points (before, immediately after, and 3 months after the intervention group received the intervention).

**Results:**

One hundred eighteen educators and 302 parents recruited from 19 participating ECEC services have consented to take part in the trial.

**Conclusions:**

There is considerable potential for ECEC services to improve everyday interactions with parents and potentially child outcomes, by implementing this practice support model. Future research in this field can examine long-term effects of improving the parent-educator relationship. The intervention has potential to be widely embedded in educator training or professional development.

**Trial registration:**

Australian New Zealand Clinical Trials Registry (ANZCTR): ACTRN12619000488101. Prospectively registered 25 March 2019.

## Contributions to the literature

- Building strong partnerships between families and early childhood educators is a valuable way to improve outcomes for children and their families
- Pre-service training in this area of partnering with parents is minimal in Australia. There is great interest from educators about practical strategies they can use to engage with parents, particularly when discussing challenging topics
- Building on a needs analysis conducted previously, we have developed a practice support system to address this need and are trialling it with ECEC services across Victoria, Australia during 2019
- Our study is an example of the evaluation of an intervention designed specifically to meet end-user needs and provide policy directions.

## Background

In their work with young children, early childhood professionals support families in many ways. Educators in early childhood education and care (ECEC) services are particularly well placed to play an important role in supporting parents^1^ to navigate common, everyday challenges in raising children. However, in an exploratory study we conducted before this project, educators told us they would benefit from greater support in how to handle challenging conversations with parents. This paper describes a cluster randomised controlled trial (cRCT) of the *Partnering with Parents* intervention that we designed to address this need.

Policymakers and the broader ECEC community have for many years acknowledged that supporting strong relationships between families and ECEC services is a powerful way to improve child wellbeing, social disadvantage, health, educational, and behavioural outcomes for children [1–6]. A reliable body of knowledge underscores the need for parental engagement in children’s learning particularly in their early years. Educators are potentially effective, non-stigmatising sources of support, who have opportunity to engage with parents on key issues related to children’s learning and development [7]. Working together, educators and parents can develop ideas for improving children’s development and wellbeing that can be applied in the home as well as in the early childhood service. There is compelling evidence that the parent-child relationship and the home environment are the most influential factors in shaping a child’s development [8–11]. The value and importance of positive relationships with families and collaborative practice in the ECEC sector is reflected in international policy and ECEC standards [12–15]. For example, in Australia the National Quality Framework for early childhood education Quality Standard 6, *Collaborative Relationships with Families and Communities*, focuses explicitly on collaboration [12]. Similarly, several of the practice principles underpinning the Victorian Early Years Learning and Development Framework (VEYLDF) highlight collaboration between early childhood staff and families as an important way to support children’s learning [13]. The VEYLDF Practice Principle *Partnerships with Families* describes the key elements that comprise a genuine partnership and is a starting point for the intervention being trialled.

In its review and recommendations about early education and care, the Australian *Lifting our Game* report [16] recognises that better parent support in the sector would be of substantial benefit. However, the pre-service training currently available in this area of partnering with parents is minimal. Before this trial, our research team conducted some preliminary exploratory research^2^ with parents and educators about their experience of partnership in ECEC settings. We found educators often felt ill-equipped for working with parents. Although educators reported they felt confident welcoming, providing information, and sharing children’s strengths, they had lower confidence and skills in raising and responding to concerns in collaborating with families experiencing vulnerability. Ninety-eight percent of 318 surveyed educators reported they would like training in working with parents. The exploratory study also investigated post-qualification professional development opportunities available to educators. This highlighted that although educators had access to various learning opportunities about working in partnership with families, the available options tended to focus on foundational knowledge and the theory of working in partnership with little or no focus on skill-building – the ‘how’ of working with parents.

To address this gap, we developed a practice support system, *Partnering with Parents*, for ECEC educators to guide them through practical strategies for working collaboratively with parents. The *Partnering with Parents* practice support system is designed to be embedded in everyday service delivery and offered in pre-service as well as in-service training courses.

A field test of *Partnering with Parents*, conducted in 2018 with five early childhood services, 50 educators and 137 parents involved trialling the resources, and refining implementation strategies, measures and the training approach. The practice support system was widely accepted by educators at the five participating services and both educators and parents gave critical feedback about their experience of *Partnering with Parents* at their service. The methodology and resources were refined based on the field test in preparation for the current experimental trial.

### Aims

This trial aims to evaluate the effectiveness of the *Partnering with Parents* practice support system by examining the following process information and intervention outcomes:

1. To what extent do educators, including educational leaders and practice coaches, use components of the practice support system? That is, which components are used, by whom, and how often? (Practice coaches are experienced educators in every service selected to assist other staff with implementation – see ‘Participants’, below)
2. To what extent do educators, including educational leaders and practice coaches, find components of the practice support system useful?
3. Following intervention, and compared with wait-list participants, to what extent are changes seen in educators’ reports of: quality of parent-educator relationships, skills and confidence in communicating with parents, and referral activities?
4. Following intervention, and compared with wait-list participants, to what extent are changes seen in parents’: measures of quality of parent-educator relationships, satisfaction with communication at the service, perceptions of the relational environment at the service, help-seeking after advice from educators at the service, and parenting confidence and parenting stress?
5. What process or demographic factors mediate or moderate the results obtained?
6. To what extent do parents’ and educators’ responses correlate for common measures?

## Methods

### Trial design

**Fig. 1:**
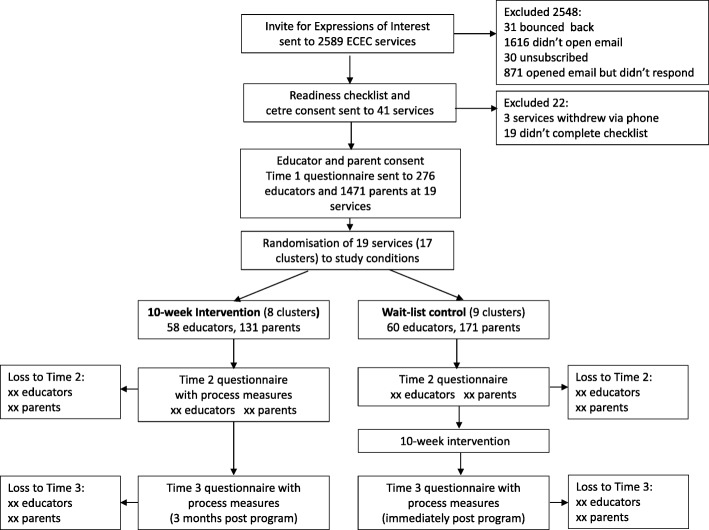
Trial flowchart. Nb. ‘xx’ represents parts of the trial that have not yet occurred at the time of writing this paper

The trial is a cluster randomised controlled trial (cRCT) with two groups, an intervention and a wait-list control group. Because the intervention will be delivered under normal service conditions, a cRCT was chosen for practical reasons and to avoid contamination of individual participant randomisation. Each service participating in the trial is a ‘cluster’ and we used stratified randomisation to match each service with another. Following this we randomly allocated one of every pair to the intervention group and the other to the wait-list control group. Details of the randomisation procedure can be found in the ‘Randomisation’ section below. The wait-list control group receive *Partnering with Parents* after the intervention (see Fig. 1 for trial flowchart). The trial was designed and will be reported in accordance with the adaptation of the Consolidated Standards of Reporting Trials (CONSORT) reporting guidelines for cluster randomised trials [17].

### Setting

ECEC has two broad service types: child care and pre-school services. In Victoria, Australia, according to the Australian Children’s Education & Care Quality Authority (ACECQA), there are 4241 ECEC services and just over 50% have an integrated kindergarten (pre-school) program with long day care. Local government or private providers operate the services with national or state funding. For this trial, we sought to recruit long day care, long day care with kindergarten, and stand-alone kindergarten ACECQA-approved ECEC services in Victoria. Using the contact details publicly available via ACECQA, two emails were sent (one in early December and one in late January) to 2589 Victorian services asking if they were interested in receiving more information about the experimental trial. We telephoned 41 sites that expressed interest and gave them information about the time commitment for implementing the intervention. During the phone call, three withdrew because of the time commitment and the remainders were sent a two-page information sheet and readiness checklist. After return of the readiness checklists, 28 services were sent a formal agreement to ‘sign up’ to trialling the intervention. Of these, 19 services returned signed agreements.

### Participants

Participants are educators and parents recruited from the 19 participating ECEC services. Out of 276 educators employed at these services at the time of recruitment, 118 gave consent to take part in the trial by completing the Time 1 questionnaire. Educators included kindergarten teachers with university (tertiary) qualifications and early childhood educators with a range of qualifications, such as certificates, diplomas and bachelor’s degrees. Services required all educators to implement the intervention whether or not they agreed to take part in the anonymous and voluntary evaluation component (the trial).

Out of 1471 families who had at least one child enrolled in one of the 19 services, 302 parents consented to take part and completed the Time 1 questionnaire.

### Eligibility criteria

Only ACECQA-approved kindergartens and long day care services in Victoria were invited to take part in the trial. Those exclusively providing out of school hours care, vacation care and family day care were excluded. Services were required to complete a Readiness Checklist – a 14-item list about their service’s capacity to implement the intervention as required. To be deemed eligible to implement the intervention a service must have responded ‘yes’ to the following critical items of the readiness checklist:

- A management structure that includes Educational Leader/s and a Coordinator
- Educators have time for professional development activity
- Educators have time for brief conversations with parents
- Educators/educational leaders/coordinator have access to the internet in work hours, and
- Someone who could take on a coaching role for educators.

At the individual level, inclusion criteria were educators and parents from participating services who consented to participate in the trial. Parents with at least one child enrolled at the service either part time or full time were eligible. One parent per child was included in the trial, and in cases where another parent completed the questionnaire about the same child, we randomly selected one of those parents for inclusion.

### Consent and recruitment

Managers from eligible services signed formal agreements to undertake the implementation of *Partnering with Parents* throughout their service and agreed to be randomly allocated to commence as a part of either the intervention or wait-list control. This formal agreement stipulated that the service would support the Parenting Research Centre (PRC) to invite individual educators and parents to complete three anonymous online questionnaires throughout the trial. However, although educators’ participation in the intervention was expected, completion of the questionnaires was voluntary and services’ management will not know which parents or educators consented to take part.

Using text suggested by the PRC, managers distributed an invitation to educators and parents in their service in various ways, including email, private social media groups, communication apps such as Storypark, and putting up a flyer at the service**.** The invitation included a link to the online plain language information statement (PLIS) and consent form. The PLIS described the study and what was involved. To consent to the study, participants read the online PLIS, clicked agreement to take part, then entered their name, email address and service into an online form. After giving consent online, participants were immediately directed to the first online questionnaire. Once they completed the questionnaire, their name, email address and service names were removed from questionnaire data and replaced by a participant code so their Time 1 questionnaire could be linked to Times 2 and 3 questionnaires. The educator and parent online consent and Time 1 questionnaire were available for a 2 week period before services were randomised and the intervention group commenced training for the implementation of *Partnering with Parents*.

### Randomisation

At the end of the Time 1 questionnaire collection period, services (clusters) were randomised to the intervention or wait-list control group. Services varied in size (i.e., number of families and staff), location, and their National Quality Standard (NQS) status and it is possible these characteristics could have an impact on outcomes (e.g., some larger services might have more resources than others). To control for possible influence of these characteristics, we used a stratified randomisation procedure to increase the similarity of the intervention and control groups. Stratified randomisation was a two-stage procedure [18]:

First, the project team matched every service with another by examining the following factors: location of service, size of service (number of staff and number of families), and their NQS status (their overall rating and their Quality Area 6 rating). This resulted in eight matched pairs as well as three small rural services grouped into one cluster, without a match. We decided to include the rural cluster in the study because their responses would still be valuable for analyses. For the purposes of randomisation for this rural cluster a ‘dummy site’ was used as their match, as the ninth pair, so that the services in the rural cluster were randomised to the intervention or wait-list control group the same way as all other services. To begin the second stage of randomisation we used the online software random.org [19] to create a random number order. An independent researcher at the PRC, who was unaware of the names or locations of services, performed the random number generation and assignment of pairs. Before the researcher began the randomisation procedure, we allocated each service an alphabetical letter. A random order of numbers from one to nine was generated, then, moving down the matched pairs list, the first random number was allocated to the first member of the first pair. This continued until the first member of every pair had a random number. Services with numbers one to five were allocated to the intervention group. Their pair was allocated to the control group. Services with numbers six to nine were allocated to the control group. Their pair was allocated to the intervention group. This process ensured each service had a 50% chance of being allocated to the intervention group.

### Blinding

Cluster RCTs are typically not blinded as intervention is usually delivered at the cluster level. In other words, throughout the trial, educator participants will know whether they are in the intervention or control group. Parent participants may or may not know, depending on whether the services choose to inform them. We are aware of this design limitation, but it is unavoidable. Researchers who are involved in training educators will know which services are in the intervention or control group. However, the researchers who analyse outcome data will be blind to intervention allocation. To facilitate this blinding, a researcher who is independent to the data analysis will create a code to identify whether a service is in the intervention or a control group. This code will not be known to the researchers conducting the analysis.

### Intervention

The *Partnering with Parents* intervention embeds evidence-based approaches to working in partnership with parents in the ECEC service. The aim of the intervention is to create an environment welcoming of and responsive to parents, and to strengthen educators’ skills and confidence to interact with parents in a way that supports their parenting.

There are three components:

- *Making moments matter*: Creating a positive relational environment every day for parents that parallels educator interactions with children and other staff. Strategies covered by this component are referred to as Warm and Gentle, Tuning In, Following Their lead, Listening and Talking, and Teachable Moments. These strategies are used incidentally in typical day-to-day interactions educators have with parents. Practice coaches use a day-by-day coaching approach to support educators to use these strategies.
- *More than moments:* Having constructive conversations with parents when needed. This is typically needed when raising a concern with a parent or responding to a parent’s concern. Practice coaches support educators to do this with just-in-time coaching*.* This involves scheduled interactions between coaches and educators to plan approaches, model and role-play strategies and reflect on actions taken.
- *Working on concerns:* This is working collaboratively with parents through a series of phases on an issue requiring more intensive, and possibly longer-term, attention. Coaching for this component is supplied by the PRC project team via a series of telephone consultations.

Implementation of the *Partnering with Parents* practice support system relies on identified practice coaches within each service supporting each other and leading their team through a 10-week schedule to introduce key concepts and strategies. Practice coaches are educational leaders, room leaders, managers or identified educators who provide specific coaching to all staff in the service on interactions with parents.

Practice coaches are supported to embed *Partnering with Parents* within their service by:

1. Initially completing a set of online e-learning tasks that introduce the components of the system, providing simulated filmed examples of parent-educator interactions and testing their knowledge with short quizzes
2. The week before the 10-week intervention begins, completing a half-day face-to-face group training session conducted by PRC trainers which focusses on *Making moments matter* and day-by-day coaching
3. Throughout the 10-week intervention, attending two group phone consultations with PRC trainers (in weeks 1 and 4) and accessing up to three individual or small group phone consultations in weeks 7–10
4. Throughout the 10-week intervention, attending further training via three webinars, held in Weeks 2, 3, and 5, which focus on *More than moments* and just-in-time coaching, as well as *Working on concerns.*
5. Being given ongoing access to the *Partnering with Parents* online portal, which houses all materials, videos and online training in the approach.

### Wait-list control

For ethical reasons we will offer the practice support system to services in the control group after the intervention group has completed the intervention. The wait period for the control group to receive the intervention is short (about 4 months after the Time 1 questionnaire) and should not be burdensome for the services waiting.

### Data collection timepoints

The following measures will be collected online via a secure data system at three time points: Time 1 = 0 weeks (before randomisation to trial conditions), Time 2 = at the end of week 10 (immediately after the intervention group has completed the intervention), and Time 3 = 3 months after the intervention group has completed the intervention and immediately after the control group has completed the intervention. Each questionnaire takes about 10–15 min to complete. To thank participants for their time, after completing the third questionnaire they will be able to download a tailored package of resources about early childhood and development produced by the PRC.

### Educator measures

#### Educator demographics (collected at time 1)

Demographic information asked of educators includes postcode of the service, age, gender, whether English is the main language spoken at home, their role at the service, number of years and/or months working in the role, how long they have worked in the ECEC sector, professional background/training, service type, how many children are in their care on a regular basis, and whether they work full time or part time.

#### Educator questionnaire (collected at time 1, time 2, and time 3)

Using a variety of response types (e.g., Likert scales, dichotomous and open text responses) the questionnaire asks educators about their relationships with parents, how they work to engage with families, their confidence in communicating with families, and whether they refer families to support when needed. The questionnaire also asks educators about their skills and confidence in their interactions, specifically where a concern was discussed. Items in the questionnaire are from a variety of sources. Some items were devised by the project team, others have been adapted from the AusParenting in Schools Teacher Survey [20], and others are from our earlier ECEC Exploratory Study.

#### Process items (collected at time 2 and time 3)

This relates to the extent to which educators and practice coaches use the support system and how it affects their views about their work. These items ask how often they are using the weekly strategies that were the focus of the intervention. Practice coaches are asked to reflect on whether the training prepared them adequately for the role. Items for the process questionnaire are designed to measure educator’s confidence and intention to create a positive relational environment using the *Making moments matter* component of the service support system.

### Parent measures

#### Demographics (collected at time 1)

Demographic information includes parent gender and date of birth and whether English is the main language spoken at home. We ask how many children they have at the service, their gender, date of birth and how many days they attend the service each week. Parents also report how long their children have attended the service, whether their children currently attend any other services, and whether they have attended another service previously.

#### Parent questionnaire (collected at time 1, time 2, and time 3)

At each time point we will ask parents about their relationship with educators, how welcome they feel at the service, how satisfied they are with the way educators communicate with them, and whether they have sought help from support services as a result of engaging with their child’s service. Items were devised by the project team and some were adapted from the Parenting Today in Victoria survey [21] conducted by the PRC, the Me as a Parent Scale [22], and the ECEC Exploratory Study.

If a parent has more than one child at the service, we ask them to select the child whose birthday is closest to the date on which they are filling out the questionnaire and respond to items with that particular child and that child’s educator as a reference point. In the case of twins or multiples, we ask parents to think of the eldest. Parents will be asked whether they have raised a concern with an educator and/or an educator has raised a concern with them, and how many times this occurred within the past 4 weeks. If this occurred, they will also be asked how satisfied they were with the interaction.

### Trial outcomes

The primary outcomes of the trial are educator confidence and skills in working with parents, and parent satisfaction with interactions with their child’s educator. Secondary outcomes include parent inclusiveness at centre, supportiveness of centre, parenting involvement with their child’s centre and learning, staff satisfaction with using *Partnering with Parents*, parent satisfaction with discussing concerns with educators, and parent psychological distress (parenting competence and confidence, parenting stress). We expect there will be greater change from Time 1 to Time 2 for the intervention group than for the control group and we expect that the change will be maintained or further improved at three-month follow-up (Time 3).

### Data analysis

Educators’ and parents’ responses to questionnaires will provide detailed data to assist us to determine whether *Partnering with Parents* is effective in increasing educators’ confidence and skills in partnering with parents. We propose a number of ways to examine the process and outcome data.

First, we will use confirmatory factor analysis using baseline data to confirm the internal validity of scales, developed using questionnaire items, to represent the outcome measures of interest. We will check commonly used goodness of fit requirements (e.g. RMSEA<.08, CFI > 0.90, TLI > 0.90) as recommended by Bryne [23]. Data for items missing completely at random will be imputed using the EM algorithm for each assessment period, with the reliability of the resulting summated scales computed using Cronbach alpha, with values of above 0.70 deemed to be acceptable.

Second, we will perform descriptive analyses of questionnaire responses to explore the nature of the data. For example, we will examine distribution properties of the scales to inform decisions about the need for any transformations before statistical analysis of between-group differences. We will also examine how participants in the trial compare with other samples, for instance, where normative data are available (such as the Parenting Today in Victoria survey results and the Me as a Parent scale data), we will compare trial participants’ responses with population norms for parents of children of similar ages.

### Baseline analysis (time 1)

As a check for appropriate balance between the control and intervention groups and to provide an overview of the study population [24], both at service and individual levels, data for the control and intervention groups will be compared at baseline. We will present the mean, standard deviation and range for continuous, approximately symmetric variables; medians, interquartile range and range for continuous, skewed variables of clusters. We will describe categorical data with percentages across sub-groups.

All statistical tests and confidence intervals will be two-sided. Between-group comparisons will be presented with 95% confidence intervals and the statistical significance level set will be at the 5% level. In addition, we will account for the clustered nature of the data for all comparative analyses to ensure correct confidence intervals and type I error rates are calculated. In addition, the intra-cluster correlation coefficient for each outcome, based on the adjusted analyses with 95% confidence interval, will be reported.

### Attrition analysis

We will calculate the proportion of missing data at the individual and cluster level. Observed and expected attrition rates will be compared for each assessment across clusters. We will not consider any data that were not available because of withdrawal of consent for data use by participants. We will use binary logistic regression to model attrition at Times 2 and 3 in terms of baseline measures and demographic characteristics. Predicted attrition probabilities for both these time points will be computed for use in completer analyses.

### Modelling strategy

Our approach to model choice is based on four main criteria: the characteristics of the data, model pre-specification informed by the previous literature, diagnostic plots and assessment of model appropriateness. We will undertake exploratory analyses of the following possible interactions to assess whether the effect of the *Partnering with Parents* practice support system intervention is modified by factors such as the experience of carers, or number of children under the educators’ care. These analyses will inform the choice of moderators for the analysis described below. An a priori model-fitting analysis strategy will also be developed to identify the order in which covariates and moderator effects are to be included in the model. We will treat services and individuals as random effects to account for the correlation of outcomes for individuals that belong to the same cluster (within cluster correlation) [25], and repeated measures of the same individual over time (within-individual correlation).

### Analysis of primary and secondary outcome measures

An intention-to-treat multi-level analysis will be conducted for each of the outcome measures with level 1 data reflecting each assessment period, level 2 data reflecting individual participants and level 3 data reflecting the various services. Changes in the outcome measures will be compared for the intervention and control groups assuming appropriate distributions for the outcome measures. Random intercepts will be assumed, while allowing for the clustered nature of the data, and adjusting for appropriate covariates as fixed effects. The model will include an indicator variable for the study groups (0 = control, 1 = intervention) as a fixed effect at level 3 and an integer time variable (1 = baseline, 2 = time2 and 3 = time3) as a random effect at level 1.

We will report the estimated intervention effect as mean outcome difference for continuous outcomes and odds ratio for binary outcomes between intervention and control groups with 95% confidence intervals and *p*-values [25]. The effect of individual moderators will be incorporated using interaction terms between time, allocated group and the individual moderators [26].

Finally, a completer analysis will be conducted with a repeated measures MANOVA analysis using inverse probability weights to adjust for any attrition bias [27]. This analysis will also account for the correlation between measures from the same participant, while allowing for the clustered trial design.

Furthermore, we will examine responses of the control group before and after receiving the intervention.

As an indication of family-centredness, we will look at items that both educators and parents completed, to compare their perceptions (e.g. whether they both agree the service is a welcoming environment for parents).

All qualitative data (open text questions) will be summarised and examined for themes. Thematic analysis is a multi-stage process involving familiarisation with the data, coding (identifying important features relevant to the research questions), searching for and reviewing themes, and defining and naming them [28].

The approach will be inductive – that is, coding and theme development directed by existing concepts or ideas. Thematic analysis will be undertaken by a researcher who has not been involved in collecting the qualitative information. To ensure generated themes accurately and consistently reflect all responses, a second researcher will independently recode responses based on themes outlined by the first researcher.

### Power analysis

The number of parents recruited for this study is 302. However, the effective sample size is less than this because we have intra cluster correlation (ICC), the value of which is unknown. The required effective sample size to detect a moderate effect size (d = 0.5) with 5% significance and 80% power is 102, which suggests that the sample size recruited is adequate as long as attrition rates are less than 50%, when the ICC is 3% (or less than 33% when the ICC is 6%).

## Discussion

Research suggests that building strong partnerships between families and early childhood educators has potential to improve health, and developmental and social factors for children and their families [1–6]. Based on our exploratory work and extensive consultation, we designed the *Partnering with Parents* practice support system to fill a gap in educator training and equip them with practical strategies they can use with parents, particularly for sensitive and important conversations. The intervention presents real-life scenarios and aims to improve the capacity of educators to work with all parents, regardless of family’s background or life circumstances. A strength of the intervention is that it will be incorporated into educators’ regular work. Services have agreed to implement *Partnering with Parents* at their service therefore educators will be using the strategies as part of their employment, thus eliminating the requirement for additional (e.g., out of work) time. The need for contextual fit has guided the intervention design. Because the resources are online, these can be accessed at times that suit educators’ individual availability and preferred access mode (i.e., mobile phone, tablet, computer).

The practice support system does not require educators to gain an exhaustive understanding of a new body of parenting-related knowledge because educators already have much to offer parents. Rather, the intervention focuses on enhancing educators’ skills and confidence in interacting with parents by providing the opportunity to practice a set of new interaction skills on a daily basis through the routine processes of their work.

Some strengths of the trial design outlined in this paper include: determining the presence of mediators of treatment effect/causal pathways, collection of relevant process data to guide future refinements; the use of matched comparison groups to reduce the differences between the intervention and control groups; and the use of a cluster design to minimise contamination effects.

A potential limitation is the use of self-report measures that require retrospective responding about the number of parent-educator interactions taking place during the intervention period. We ask participants to provide counts of conversations they’ve had during the past 4 weeks, which may result in recall bias. We have endeavoured to minimise the inaccuracy of these results by asking educators to keep count as they go, but this is not possible for parent data so we will rely on their memory of these interactions and their ratings of them. Also, some educators and practice coaches may have underestimated the time required to adequately teach and implement the practice strategies and may not spend the required time on each aspect of the practice support system throughout their busy days. To examine this we will collect process data via self-report measures and e-user statistics of the online profile where the resources are accessed to ascertain which and how many of the intervention resources educators accessed.

Another possible limitation is that educators may rate themselves highly on measures before they receive the intervention, and then redefine their standards after being exposed to the strategies and definitions in the intervention. This could mean that the differences between pre- and post-intervention outcomes are understated. Furthermore, the intervention and questionnaires are only available in English, yet we do not assess whether participants can read sufficient English to complete the questionnaires. Therefore, we will not know the extent to which participants not proficient in English needed assistance to complete questionnaires or to understand the intervention content.

Access to the intervention and questionnaires requires ECEC educators and parents to have an email address and the capacity (computer or phone) to access questionnaires online. Although the internet is an efficient way to reach most families, we may be missing valuable feedback from those who did not have access to the internet during our short (2-week) recruitment period. Another consideration for recruitment time is that a small number of services (kindergartens) were starting school holidays in the second week of our recruitment, so may not have been checking emails at this time. This may have affected the recruitment rate for the educators and parents from these types of centres.

In conclusion, there is considerable potential for ECEC services to improve everyday interactions with parents and potentially child outcomes as a result of implementing this practice support model. Future research in this field can examine longer term effects of improving the parent-educator relationship on the wellbeing and learning outcomes of children. The intervention has potential to be widely embedded in educator training or professional development.

### Trial status

Ongoing. The intervention group has completed the intervention. Time 2 data is currently being collated, and follow-up data is yet to be collected.

## Data Availability

Data sharing is not applicable to this article as no datasets have been analysed at this time. Participant consent does not include seeking permission for data to be made publicly available. Inquiries about the data management, questionnaires and intervention materials should be directed to the Parenting Research Centre www.parentingrc.org.au

## Abbreviations

ACECQA: Australian Children’s Education & Care Quality Authority
ANZCTR: Australian New Zealand Clinical Trials Registry
CONSORT: Consolidated Standards of Reporting Trials
ECEC: Early Childhood Education and Care
NQS: National Quality Standard
PLIS: Plain Language Information Statement
PRC: Parenting Research Centre
RCT: Randomised Controlled Trial
VEYLDF: Victorian Early Years Learning and Development Framework

## Footnotes

1 Please note that the term ‘parent’ is intended in an inclusive way to refer to the person who is in the ‘parenting role’ (i.e., mother, father, other care), regardless of their biological relationship to the child. We also use the term ‘parenting’ to refer to what parents do when fulfilling the task of nurturing and caring for children.

2 Parenting Research Centre. Exploratory report: collaborating with families: practice support model for the early childhood education and care sector. Unpublished report, Melbourne, Australia. 2016.
